# The Nomadic Digital Pathologist. Validation of a simple, dual slide scanner with remote reporting for a regional upper gastrointestinal specialist multidisciplinary meeting

**DOI:** 10.1016/j.jpi.2022.100161

**Published:** 2022-11-26

**Authors:** Tim S Bracey

**Affiliations:** Royal Cornwall Hospital, Treliske, Truro TR1 3LJ, UK

**Keywords:** Cancer multidisciplinary team, Remote diagnosis, Digital pathology, Whole Slide Imaging, UK cancer networks, NHS, Esophagus, Stomach, Cancer, Histopathology, Cell biology, Dysplasia, Carcinoma, Specialist, COVID19

## Abstract

*Background:* This article describes how a simple slide scanner with remote viewing software enabled a remote “nomadic” pathologist to continue his role as specialist lead for a regional gastrointestinal multidisciplinary team meeting (MDTM) after relocating to another site in the 5 hospital Southwest UK Peninsula cancer network just prior to the COVID-19 pandemic.

*Materials and methods:* The author used digital pathology (DP) to supplement a conventional workflow as a way of minimising delay in reporting and reviewing slides for a regional specialist Oesophagogastric MDTM (the OGSMDT). The specialist centre at University Hospital Plymouth (UHP) is 58 miles from the author’s new workplace at Royal Cornwall Hospital (RCHT). Slides from the 44 cases (10% of this specialist annual workload) in this validation study were reported or reviewed digitally using the slide scanner. All were listed for the OGSMDT due to being clinically suspicious for upper gastrointestinal malignancy, having been processed at UHP, or one of the other hospitals in the cancer network.

*Results:* The scanner allowed the author who was only on site at UHP 1 day per week to prevent delays in reporting/reviewing glass slides, using remote DP. Confidence in digital diagnosis was assessed using the Royal College of Pathologists recommendations. The author was the primary pathologist signing out 31, and second opinion for the remaining 13 cases. These comprised a mixture of biopsies as well as endoscopic and surgical excision specimens. The DP system enabled the author to report the cases digitally with an equivalent degree of confidence to glass slides and no significant discrepancies were identified between the author’s digital and final glass slide diagnosis.

*Conclusions:* The scanner was found to be safe and effective for remote reporting and review for OGSMDT cases. It was recognised that DP was advantageous to enable this role to continue remotely but that a fully integrated digital reporting system capable of high-capacity scanning would be preferable to the simple system used.

## Introduction

Digital pathology (DP) is the general term used for the scanning of glass slides to produce high quality digital images in histopathology.^1^ Previous international studies have demonstrated non-inferiority of DP whole slide images (WSI) compared with a conventional workflow,^2^^,^^3^ but widespread adoption of this technology in the UK has been slow despite advantages demonstrated by early adopters.^4^^,^^5^ Expensive equipment, ongoing subscription, and digital storage costs may be difficult to justify in DP business cases, but since home and remote working has become more commonplace, the argument for implementation of this technology is more persuasive.

**Fig. 1:**
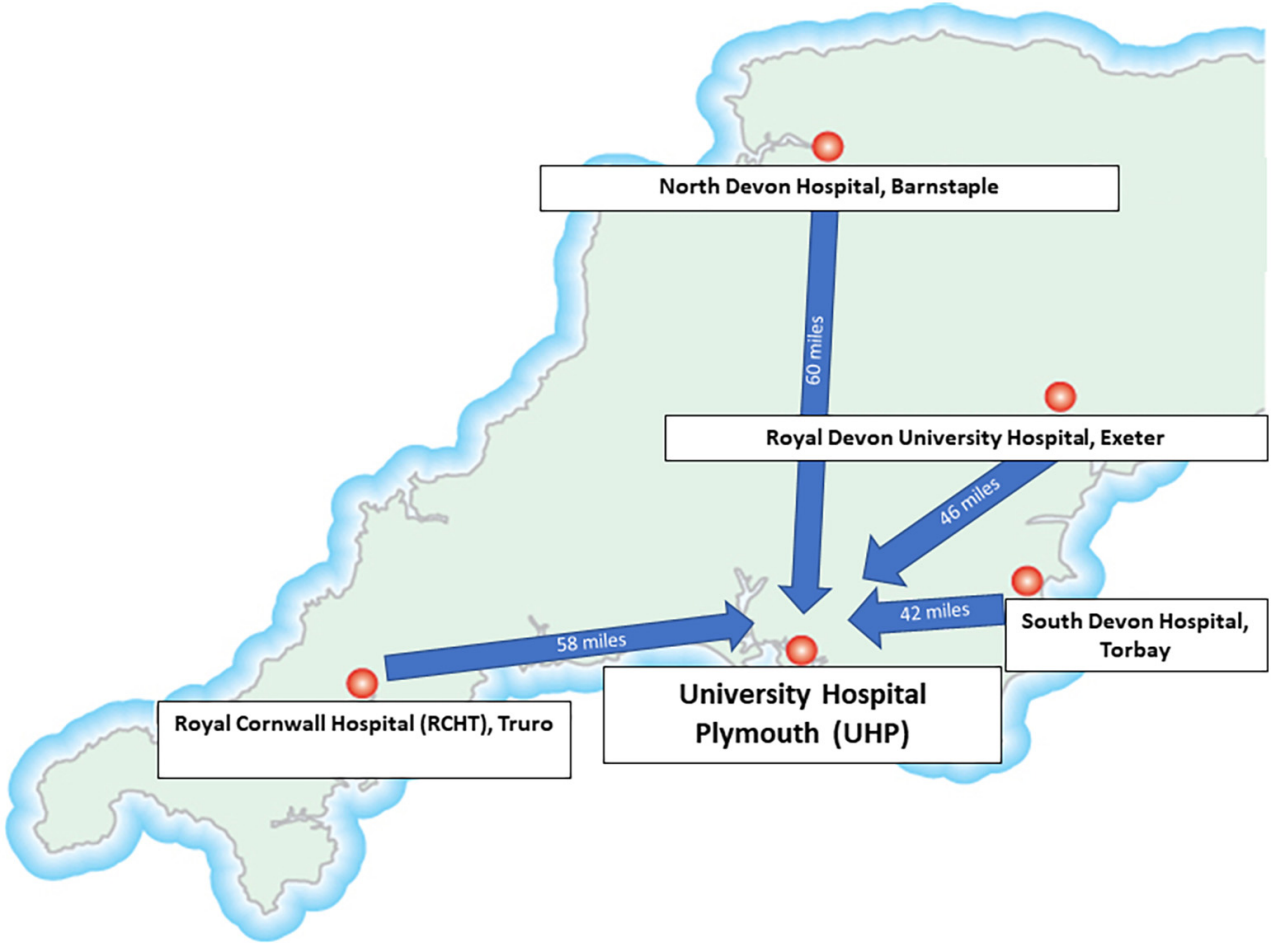
The OGSMDT takes place at UHP, Derriford Plymouth, which is the specialist centre for this surgical service in the South West UK Peninsula Cancer network. The other hospitals are between 42 miles (South Devon) and 60 miles (North Devon) from the specialist centre. Digital pathology has the potential to reduce delays relating to the distance between the hospitals in this geographically dispersed network which are among the only large hospitals in the UK not linked by any motorways and suffer from considerable traffic delays especially during the holiday season.

The OGSMDT takes place at University Hospital Plymouth (UHP) each Thursday, and involves discussion of the clinical, endoscopic, pathology, and imaging findings relating to patients from the 5 South West England Peninsula hospitals (UHP, RCHT, Exeter, North Devon in Barnstaple, and South Devon in Torbay). The other 4 hospitals have their own local MDTs, listing their suspected or confirmed oesophageal and gastric cancer patients for the OGSMDT once basic diagnostic workup is complete. While most of the diagnostic workup is performed locally at the peripheral hospital, tissue diagnoses and pre-treatment staging are expected to be finalised at the OGSMDT prior to definitive oncological and or surgical management. All radical surgery is performed at UHP, and there is consequently considerable time pressure to prepare and transport the relevant material and information from the peripheral hospitals (situated between 42 and 61 miles away; at least 50- to 95-min drive without traffic) to enable prompt MDT discussion and surgical planning (see Fig. 1).

The author had been the regional pathology lead for OGSMDT since 2011 while working full time at UHP. On relocating to Royal Cornwall Hospital (RCHT) in 2018 from UHP, the author was supported by both employers to continue the specialist role, being allocated job planned time on site at UHP with additional remote programmed activity allowance. Since the OGSMDT had already been taking place via a teleconferenced link for many years to enable remote regional core members to take part, this transition was seamless as there was no necessity to be physically present at UHP for the meeting itself. The author works onsite at UHP each Tuesday afternoon, 2 days prior to the MDT. With a conventional glass slide workflow however, this meant some cases would have to wait until at least the following week to be authorised or reviewed depending on the timing of processing, allocation, and necessity for additional lab work. Digital pathology offered obvious advantages to the author by removing the necessity to be physically present when slides either arrived from the peripheral hospitals or finished processing at UHP at times when working remotely from the specialist centre. The small, single, and dual slide scanner supplemented rather than replaced the conventional glass slide workflow, being particularly beneficial at times during the COVID-19 pandemic when onsite working was discouraged.

Despite being involved in a recent feasibility study of multisite DP reporting in the UK,^6^ the author is not aware of any previous publication where DP has been used specifically to facilitate a multiple hospital-site, remote specialist cancer multidisciplinary team (MDT) meeting.

This publication also serves as a record of the author’s self-validation process carried out during a period of exceptional service pressure, according to guidance from the UK Royal College of Pathologists.^7^^,^^8^

## Background and methods

The author has developed a method for conducting histology reviews prior to the OGSMDT which involves communication (via a secretary at UHP) with the specialist nurse and MDT coordinator on a Tuesday morning regarding the cases provisionally listed for the MDT. The author reviews the details of any cases, including those already authorised, and chooses which of the external cases are likely to need formal review, and any diagnostic or post-operative cases that still require primary reporting. Many of these are already available at UHP, and glass slides will be reviewed or reported at the time of visiting but cases often miss the Tuesday afternoon deadline. When working remotely, and when slides fail to arrive at UHP in time, slides are selected for digital scanning.

A dual slide/mega-slide (Objective Imaging Glissando) WSI (whole slide image) scanner was installed at UHP in September 2019 to support the UHP medical liver service. Agreement for the author to use the device for the OGSMDT was established following negotiation with information technology (IT) and information governance (IG) at UHP. The author was given a laptop by UHP to enable continued access to the UHP laboratory information management system (LIMS) via a virtual private network (VPN). Although IG approval prohibited sharing slides with the original slide label (which contained part of the patients’ name), the scanner was able to recognise the 2D barcodes on the UHP glass slides, automatically assigning the case number to each slide, enabling the correct case to be identified and retrieved by accessing the LIMS.

The scanner was used in conjunction with “Microvisioneer” software, which enabled a folder to be shared with a hyperlink. The folder hyperlink was saved into the author’s internet bookmarks, enabling any new cases to be easily seen through a simple web link, provided there was direct connection to the UHP local area network (possible from within UHP, or the RCHT pathology department) or remote access from home via VPN. After authorisation of the pathology report, and checking of glass slides at UHP, the WSI slide files were transferred to the internal department server (the .svs filename suffix was automatically assigned to the laboratory case number). The author used dual 24” consumer grade monitors at work, and 27” gaming quality monitors at home. The author used the “Point-of-Use Quality Assurance Tool for Digital Pathology Remote Working”^9^ with all monitors to ensure the displays were sufficient and comparable.

The author was already an experienced user of DP and had previously viewed a selection of practice cases prior to this “stage 2” validation project. At the time of the pandemic when updated guidance was issued from the Royal College of Pathologists,^7^ it was considered appropriate to begin reporting live cases digitally using this system promptly to ensure that delay to OGSMDT cases was minimised. Most cases however were still reported and reviewed onsite at UHP using a conventional glass slide workflow, and most cases were shown to another pathologist prior to authorisation. The 44 cases documented in Table 1 below represent cases that were primary reported or reviewed remotely as a second opinion to prevent unnecessary delay. They are enriched for urgent cases and do not necessarily represent a true representation of the average case mix in this sub-speciality. Confidence in the digital diagnosis was approximated using the Likert psychometric scale (1=not confident at all, 7=very confident) recommended by the Royal College of Pathologists^8^^,^^10^^,^^11^ for digital pathology self-validation.

**Table 1:** Of the ∼400 cases reported and reviewed for the OGSMDT per year, these 44 cases were recorded as having been reported or reviewed using the DP system.

Case	Clinical site	Specimen type	History	Histological diagnosis	Primary diagnosis or Second opinion	Digital confidence (1–7)
1	Stomach	Biopsy	Malignant endoscopy	Poorly differentiated adenocarcinoma	Primary diagnosis	7
2	Oesophagus	Biopsy	Suspicious endoscopy	Benign inflammatory	Primary diagnosis	6
3	Stomach	Biopsy	Malignant endoscopy	Poorly differentiated adenocarcinoma	Second opinion	7
4	Stomach	Biopsy	Malignant endoscopy	Poorly differentiated adenocarcinoma	Primary diagnosis	7
5	Stomach	Biopsy	Abnormal CT	Poorly differentiated adenocarcinoma	Second opinion	7
6	Oesophagus	Biopsy	Suspicious endoscopy	Poorly differentiated adenocarcinoma	Primary diagnosis	7
7	Oesophagus	Biopsy	Suspicious endoscopy	Squamous cell carcinoma	Primary diagnosis	7
8	Oesophagus	Biopsy	Barrett's surveillance	Low-grade dysplasia with aberrant p53	Primary diagnosis	5
9	Oesophagus	Biopsy	Suspicious endoscopy	Squamous cell carcinoma	Primary diagnosis	7
10	Stomach	Biopsy	Suspicious endoscopy	Mucosa only shows gastropathy changes	Second opinion	5
11	Stomach	Endo-scopic excision	Nodule in Barrett's EMR	pT1a adenocarcinoma	Second opinion	6
12	Oesophagus	Biopsy	Barrett's surveillance	HGD with some suspicious features	Second opinion	6
13	Stomach	Biopsy	Abnormal imaging	Helicobacter gastritis	Second opinion	6
14	OG junction	Endo-scopic excision	Nodule in Barrett's EMR	pT1a adenocarcinoma	Second opinion	7
15	Stomach	Biopsy	Persistent HGD unfit for op	HGD with some suspicious features	Second opinion	6
16	Stomach	Surgical resection	Oesophagectomy for adenocarcinoma	Amyloid in ectopic pancreas	Second opinion	7
17	Oesophagus	Surgical resection	Oesophagectomy for adenocarcinoma	pT1a adenocarcinoma	Second opinion	7
18	Stomach	Biopsy	Linitis plastica for molecular testing	Poorly differentiated carcinoma	Second opinion	6
19	Stomach	Biopsy	External suspicious biopsy for second opinion	High grade dysplasia with some suspicious features	Second opinion	6
20	Stomach	Wedge excision	Suspected GIST gastric polyp	Well differentiated neuroendocrine tumour	Second opinion	6
21	Oesophagus	Biopsy	Malignant endoscopy	Poorly differentiated adenocarcinoma	Primary diagnosis	7
22	Oesophagus	Biopsy	Malignant endoscopy	Poorly differentiated adenocarcinoma	Primary diagnosis	7
23	Stomach	Biopsy	Suspicious endoscopy	HGD with some suspicious features	Primary diagnosis	7
24	Stomach	Biopsy	Suspicious radiology	Normal limits	Primary diagnosis	7
25	Oesophagus	Surgical resection	For cancer	Staging finalised	Primary diagnosis	7
26	Oesophagus	Biopsy	External biopsies of nodule for EMR	Ulcerated adenocarcinoma	Primary diagnosis	7
27	Oesophagus	Biopsy	Barrett's surveillance	Not dysplastic	Second opinion	7
28	Stomach	Biopsy	gastric polyp biopsies query pyloric adenoma	Not dysplastic	Second opinion	6
29	Stomach	Biopsy	Gastric mass query linitis	MALT lymphoma	Second opinion	5
30	OG junction	Biopsy	Suspected malignant	Benign ulcer	Primary diagnosis	5
31	Lower oesophagus	Biopsy	Suspected malignant	Benign ulcer	Primary diagnosis	5
32	Oesophagus	Biopsy	Suspicious endoscopy	Adenocarcinoma	Primary diagnosis	7
33	Stomach	Biopsy	Malignant endoscopy	Adenocarcinoma	Primary diagnosis	7
34	Oesophagus	Biopsy	External endoscopic biopsy for OGSMDT review	Squamous cell carcinoma (acantholytic variant)	Primary diagnosis	7
35	Oesophagus	Biopsy	Malignant	Squamous cell carcinoma	Primary diagnosis	7
36	OG junction	Biopsy	Suspicious endoscopy	Adenocarcinoma	Primary diagnosis	7
37	Oesophagus	Biopsy	Suspicious lesion at Varices surveillance	Squamous cell carcinoma	Primary diagnosis	7
38	Stomach	Surgical resection	gastrectomy for large junctional SIII adenocarcinoma	Staging finalised	Second opinion	7
39	OG junction	Biopsy	Suspicious endoscopy and imaging	Poorly differentiated adenocarcinoma signet ring mucinous	Primary diagnosis	7
40	OG junction	Biopsy	Suspicious endoscopy referred for EMR	High grade dysplasia no suspicious features	Primary diagnosis	7
41	Stomach	Biopsy	Suspected linitis plastica	Normal mucosa. Levels and Cytokeratin requested	Primary diagnosis	6
42	OG junction	Biopsy	Malignant endoscopy but previous biopsy HGD only	Adenocarcinoma	Primary diagnosis	7
43	OG junction	Biopsy	External LGD but endoscopic suspicious lesion	Adenocarcinoma (at least pT1a) on review	Primary diagnosis	7
44	Stomach	Biopsy	Distended stomach peritoneal mets	Atypical cells not diagnostic	Primary diagnosis	6

## Results

Previous UHP internal (unpublished) audits have shown that 250–300 first diagnosis of neoplasia are reviewed annually in the OGSMDT, with two-thirds of this number being from outside of UHP. Up to 100 radical specimens are submitted by our specialist surgical team each year and are all processed and reported at UHP. An additional 20–30 endoscopic resections (EMR) are done each year at UHP, and all the invasive cases are reviewed at the OGSMDT. This amounts to approximately 400 cases per year, of which the 44 cases below comprise around 10% of this specialist workload.

The author was the primary pathologist signing out 31 of 44 cases, and second opinion for the remaining 13 cases. Some of the external cases were considered primary diagnoses (at the specialist centre) although they were effectively reviews of previously diagnosed cases.

The cases comprised a mixture of biopsies as well as endoscopic and surgical excision specimens. Most cases consisted of 1–2 slides, and cases comprising more slides usually represented 1 or 2 “key slides” to complete diagnosis and/or staging remotely following additional lab work. Although the scanner has a fast scan speed (less than 3 min per endoscopic biopsy slide) only 1–2 slides could be loaded at a time and depended on availability and good will of the busy laboratory staff. Urgent cases with fewer slides were therefore prioritised for scanning on the day of the MDT by referring to the schedule issued to all core members. The case mix was clearly biased towards malignancy with the OGSMDT being a specialist cancer meeting and most cases being chased just prior to the meeting were generally straightforward cancers which were easy to diagnose digitally. Some cases chosen for digital review also included those where the pathology was unusual, or discordant with clinical expectations. For example, 5 cases were clinically suspicious but appeared benign on histology. These were all signed out on the glass slides to ensure the remote DP diagnosis was correct.

**Fig. 2:**
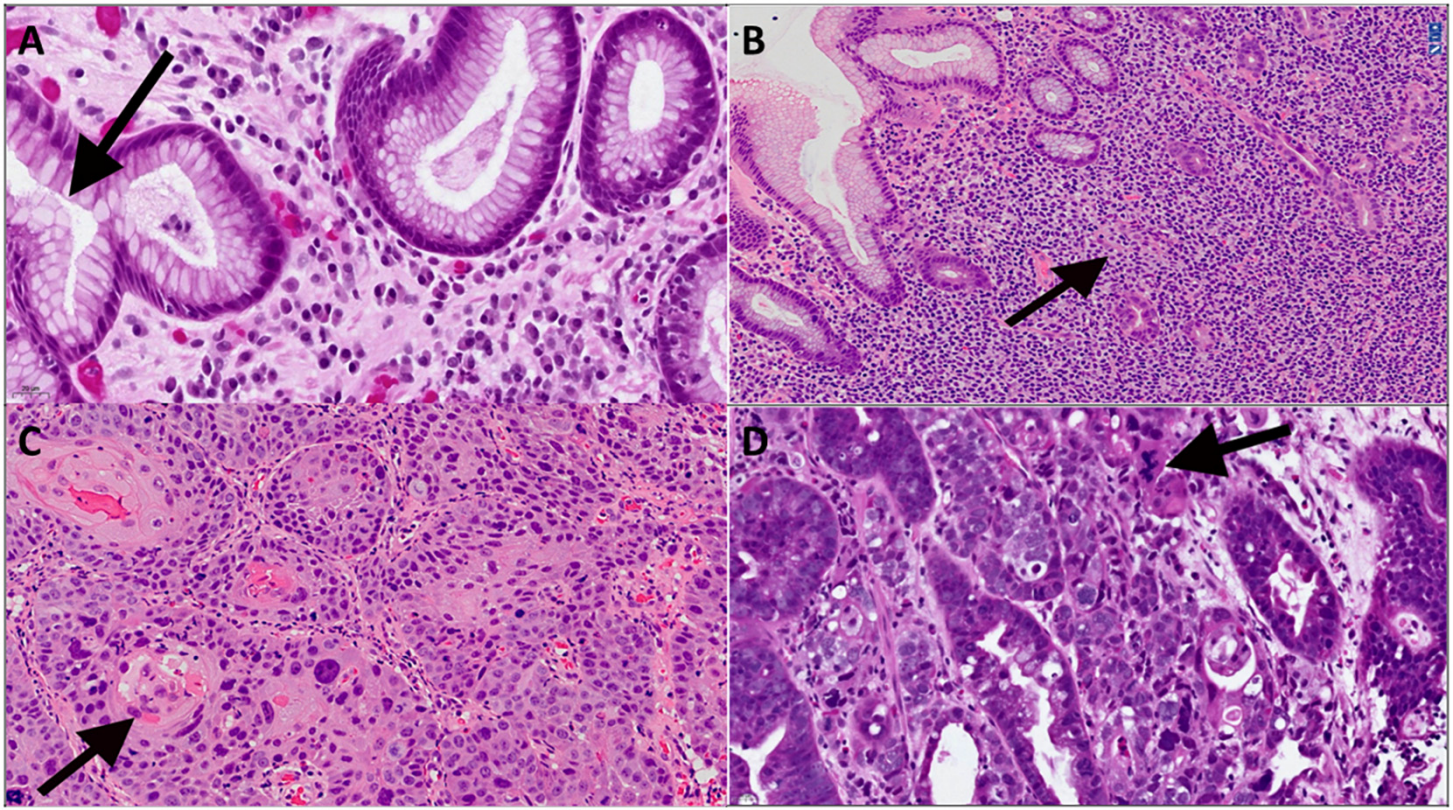
(A) Case 13: Helicobacter gastritis (arrow = small numbers of *H. pylori* organisms), (B) Case 29: MALT lymphoma (arrow = lymphoepithelial lesion), (C) Case 37: Squamous cell carcinoma (arrow = keratin pearl), (D) Case 17: Poorly differentiated adenocarcinoma in a small area of gastric polyp initially diagnosed outside the specialist centre as adenoma with low-grade dysplasia (arrow = atypical mitosis). This discrepancy was identified by the author on the digital slide.

Despite confidence in assessing images on the DP system, the benign cases were recorded as lower confidence diagnoses (5 or 6 out of 7). These and other non-carcinoma cases were difficult, not due to DP, but due to an apparent conflict between the histology features and the clinical impression. There was a consequent tendency therefore, for these cases to be recorded as a lower confidence level and require additional lab work. For example, Case 13 showed Helicobacter gastritis (Fig. 2D) despite having abnormal cross-sectional imaging of the stomach, Case 20 was a neuroendocrine tumour (suspected to be a GIST clinically), and Case 29 was a (MALT) lymphoma rather than cancer as suspected clinically. With DP, the author was not only able to sign out cases between visits to UHP but was also able to collaborate with colleagues who could look at the glass slides on site at UHP after they had been scanned. There were no significant discrepancies identified between the author’s digital impression and the glass slide diagnosis.

Most of the cases scanned were either carcinomas or precancers (dysplasia). In keeping with previous local data, approximately 80% of the invasive cancers with adenocarcinoma with the remainder being squamous cell carcinoma (eg. Fig. 2C) and undifferentiated carcinoma. Four cases were suspicious but not diagnostic of invasion. Of the dysplastic cases, one from another hospital in the network was found to harbour a small focus of adenocarcinoma on remote digital review (case 17; Fig. 2D). This diagnosis was made with confidence digitally but was also confirmed on the glass slide and shown to colleagues before final sign out. The rapid and confident digital review however enabled a prompt provisional verbal opinion at the time of the MDT and an appropriate change in management plan.

Table 2 outlines the proposed advantages and disadvantages of the system used in this validation, compared with a conventional workflow and the possibility of a fully integrated DP system of the type desperately needed for truly efficient cross-site specialist histopathology working.

**Table 2:** The author’s opinions on the advantages and disadvantages of the Glissando Microvisioneer DP system used in this validation.

Advantages	Disadvantages
- Simple web browser connection via local area network (LAN).	- Viewing scans outside the LAN requires a VPN which considerably slows slide loading.
- Good 40x scan quality with very low rescan rate	- Scanning is not completely automated and only one or two slides can be scanned at a time
- Colour balance is clear and vivid	- Colour balance is different (more intense) than the original glass slide
- System is secure and confidential	- Because slide labels were not allowed. the potential of mixing up one patient's sample with another remained, as names are usually one of the checks a pathologist makes when looking at a slide
- Remote viewing window allows smooth scrolling towards mouse pointer.	- Remote window does not have a thumbnail with which to "drive" the slide so the panning is not as quick or intuitive as fully integrated DP systems.
- No software install needed at remote site (web browser only)	- No other annotations available. no potential for snapshots or saving annotations or downloading WSIs across network connection in contrast to some other systems.
- Multiple cases can be viewed separately in the same archive	- No ability to view slides from the same case side by side to view for example IHC next to H&E slides
- Slide label is scanned automatically by the scanner	- LIMS must be used in a separate window from DP images (report and clinical information which would ideally be viewed in the same system).

It was immediately apparent at the beginning of this study that the scanned slides had a different colour intensity to the original glass slide. Although this did not reduce confidence in making a simple diagnosis, it did require some familiarisation. In contrast to the findings of some earlier studies 12, the author did not perceive any difficulty in recognising and grading dysplasia using DP. The biggest difference in appearance of WSI was related to the differences in staining and section thickness of the original glass slides between the different network hospitals. The author has had more than a decade of experience in adapting to these differences in H&E appearance between the 5 hospitals and it was therefore not perceived as a specific limitation of the DP system. In keeping with published studies, increased use of p53 immunohistochemistry has improved diagnostic concordance in Barrett's oesophageal dysplasia, and the author finds p53 staining useful in DP to overcome variations in H&E section appearance between the different Cancer network laboratories.^11^

More significant limitations of the system compared to a sophisticated DP image management system included the lack of integration with the LIMS and lack of automation. A significant potential advantage of fully integrated DP is related to the safety of reporting into the same program, as the images are being viewed, preventing potential mismatches. Additional care was needed with this pilot study to ensure that the slide number matched, since the original label could not be viewed due to local IG restrictions. In addition, since the slides were viewed within a browser window rather than being opened in a specific DP viewer, more advanced multiple slide functions, measuring and labelling were not available. Glass slide postage was still required for occasions for cases from the other 3 hospitals in the Peninsula network. At the time of writing, South Devon, North Devon, and Exeter did not have a DP system, and the author did not have access to these other 3 separate laboratory IT systems.

Accepting the above limitations; however, the Glissando-Microvisioneer slide sharing system was easy to use, secure, and confidential and did not require any specific software installation, and therefore would be acceptable to many pathologists looking for a way of viewing microscope slides quickly and efficiently when a full DP system is unavailable in their place of work.

## Discussion

The recent COVID19 pandemic has highlighted the advantages of remote working when staff and family members are required to isolate or reduce physical interaction with colleagues even when fit to work.^12^ Histopathologists are increasingly compelled to specialise in order to offer a quality service and keep up to date with clinical scientific progress. The Southwest UK Peninsula hospital network is geographically dispersed (Fig. 1), and several of the cancer MDTMs are centralised, necessitating transport of material and reports between different organisations with ring-fenced IT systems, leading to the potential for delays to the diagnosis and management of cancer patients. Although previous studies have examined the use of DP across cancer networks, in tertiary centres, local tumour boards, and for frozen sections,^6^^,^^13^, ^14^, ^15^ the author is not aware of another publication documenting the use of a small slide scanner to support a remote regional expert histopathologist to continue a lead role in a multisite specialist MDTM. This simple single- and dual slide scanner with remote viewing software was used to validate slides from 44 cases, comprising a tenth of the author’s annual OGSMDT workload. Not only were no significant discrepancies identified between the digital impression and final glass diagnosis but the author identified a focus of malignancy on remote digital review in a lesion initially reported externally as a benign adenoma, resulting in an immediate and significant change to patient management.

Continued access to a simple and relatively inexpensive method of remote reporting may be a considerable advantage to pathologists in the future, particularly those anticipating developing a sub-speciality role with flexible working patterns. Developing expertise in a histopathology sub-speciality is accelerated by interaction with an expert clinical team and a large and varied case load deriving from multiple hospital sites.^11^ The author has been a regional pathology lead for the OGSMDT at UHP for many years and wished to continue this role on relocating to one of the other Peninsula hospitals (RCHT). Whilst it is not uncommon for a senior consultant to relocate to a smaller institution during their career, a general hospital may not anticipate benefiting from their specialist knowledge and skills. The author has been fortunate to relocate to a smaller hospital in the same cancer network but continue a specialist diagnostic role and maintain knowledge and skills. Remote working in histopathology can of course be enabled with postage of slides between different hospitals. Transfer of glass slides between separate hospital trusts however can lead to significant delays when additional administration with booking-in and tracking of material between different hospital sites, and when different computer systems and protocols are factored into the process.

The Peninsula OGSMDT requires communication between health care professionals in 5 separate hospital trusts in a dispersed geographical region. The simple slide scanning method used in this validation study was based at the UHP specialist centre. Currently slides from outside of the author’s employing trust are sent to UHP and only scanned when requested, but ideally all the 5 trusts would have slide scanning facilities and optimally would have the same instance of a laboratory information management system (LIMS). This would allow instantaneous access to digital images of slides scanned at all 5 sites and minimise the current delays in transporting material to the specialist centre. The benefits the author has experienced using this simple DP system could therefore be more widely shared to the benefit of the wider consultant group, trainees, and other regional subspeciality experts.

This more sophisticated DP system is in the planning process in this region and would clearly enable a more seamless remote reporting and specialist MDTM process. Nonetheless, this study has demonstrated that most cases in this regional specialist MDTM can be diagnosed with confidence remotely using a simple DP system. It is hoped that the advantages demonstrated by the simple slide scanning and reporting system described here will catalyse a more widespread DP rollout in our region. Furthermore, by embracing modern digital reporting technology we hope to witness an improvement in the reputation and recognition of the critical importance of histopathology in cancer diagnosis. Consequently, increased future recruitment and investment in digital cellular pathology services will eventually translate into improved cancer treatments and patient outcomes.

## Declaration of Competing Interest

The authors declare that they have no known competing financial interests or personal relationships that could have appeared to influence the work reported in this paper.
